# Real world study of sacituzumab govitecan in metastatic triple-negative breast cancer in the United Kingdom

**DOI:** 10.1038/s41416-024-02685-9

**Published:** 2024-04-24

**Authors:** Daire Hanna, Sophie Merrick, Aruni Ghose, Michael John Devlin, Dorothy D. Yang, Edward Phillips, Alicia Okines, Neha Chopra, Elisavet Papadimatraki, Kirsty Ross, Iain Macpherson, Zhuang Y. Boh, Caroline O. Michie, Angela Swampillai, Sunnia Gupta, Tim Robinson, Lewis Germain, Chris Twelves, Charlotte Atkinson, Apostolos Konstantis, Pippa Riddle, Nicola Cresti, Jay D. Naik, Annabel Borley, Amy Guppy, Peter Schmid, Melissa Phillips

**Affiliations:** 1 St. Bartholomew’s hospital, Barts Health NHS trust, London, UK; 2 Barts Cancer Institute, Queen Mary University, London, UK; 3 UCLH NHS foundation trust, London, UK; 4 The Royal Marsden NHS foundation trust London and Sutton, London, UK; 5 The Royal Free, London NHS foundation trust, London, UK; 6 Beatson West of Scotland Cancer Centre, Glasgow, UK; 7 Edinburgh Cancer Centre and University of Edinburgh, Edinburgh, UK; 8 Guy’s and St Thomas’ NHS foundation trust, London, UK; 9 Bristol Haematology and Oncology Centre- NHS foundation trust, Bristol, UK; 10 Leeds Cancer Centre, Leeds, UK; 11 Taunton and Somerset NHS foundation trust, Taunton, UK; 12 Princess Alexandra Hospital NHS trust, London, UK; 13 Chelsea and Westminster, London, UK; 14 Northern Centre for Cancer Care, Newcastle upon Tyne Hospital NHS Foundation Trust, Newcastle upon Tyne, UK; 15 Harrogate and District NHS foundation trust, Harrogate, UK; 16 Velindre Cancer Centre, Cardiff, UK; 17 Mount Vernon Cancer Centre, London, UK

**Keywords:** Breast cancer, Breast cancer

## Abstract

**Background:**

Treatment options for pre-treated patients with metastatic triple-negative breast cancer (mTNBC) remain limited. This is the first study to assess the real-world safety and efficacy of sacituzumab govitecan (SG) in the UK.

**Methods:**

Data was retrospectively collected from 16 tertiary UK cancer centres. Pts had a diagnosis of mTNBC, received at least two prior lines of treatment (with at least one being in the metastatic setting) and received at least one dose of SG.

**Results:**

132 pts were included. Median age was 56 years (28–91). All patients were ECOG performance status (PS) 0-3 (PS0; 39, PS1; 76, PS2; 16, PS3;1). 75% (99/132) of pts had visceral metastases including 18% (24/132) of pts with CNS disease. Median PFS (mPFS) was 5.2 months (95% CI 4.5–6.6) with a median OS (mOS) of 8.7 months (95% CI 6.8-NA). The most common adverse events (AEs) were fatigue (all grade; 82%, G3/4; 14%), neutropenia (all grade; 55%, G3/4; 29%), diarrhoea (all grade; 58%, G3/4, 15%), and nausea (all grade; 38%, G3/4; 3%). SG dose reduction was required in 54% of pts.

**Conclusion:**

This study supports significant anti-tumour activity in heavily pre-treated pts with mTNBC. Toxicity data aligns with clinical trial experience.

## Background

In the UK, approximately 8000 people are diagnosed with TNBC every year, accounting for 15% of all breast cancer cases [1], and carrying the poorest prognosis compared with other subtypes [2]. Estimates of median overall survival (mOS) in the metastatic setting vary but is approximately 18 months [3]. TNBC is an extremely heterogeneous disease and lags significantly behind other subgroups in the development of targeted treatments [4–6]. Prior to the approval of SG, the current standard of care relied on chemotherapy [5–7]. Programme death ligand 1 (PD-L1) inhibitors are given in combination with chemotherapy for approximately 30–40% of patients with TNBC who are considered PD-L1 positive [8–11].

SG is an antibody-drug conjugate (ADC) comprising of the humanised anti-TROP2 (trophoblast antigen 2) antibody conjugated to SN-38, the active metabolite of irinotecan, via a hydrolysable linker [12, 13]. TROP2 is a transmembrane protein which is overexpressed in multiple cancers including TNBC [14]. The antibody component binds to TROP2, facilitating the internalisation of SN-38 and eliciting its anti-tumour effects upon hydrolysis of the linker [15].

ASCENT (NCT02574455) is a phase 3 randomised clinical trial comparing SG with chemotherapy of physician’s choice (eribulin/vinorelbine/capecitabine/gemcitabine) in the relapsed or refractory mTNBC setting. SG demonstrated benefit in survival outcomes compared to chemotherapy, with a median progression-free survival (mPFS) of 5.6 (95% CI 4.3–6.3) vs 1.7 (95% CI 1.5–2.6) months and mOS of 12.1 (95% CI 10.7–14) vs 6.7 months (95% CI 5.8–7.7) [16]. This led to FDA approval of SG in early April 2021 for unresectable locally advanced or mTNBC who had received at least 2 lines of prior systemic therapy, one of which needed to have been delivered in the metastatic setting. Subsequently, in August 2022 NICE approved SG in patients with mTNBC following two lines of treatment, with one line required in the metastatic setting.

## Method

Data was retrospectively collected from 16 tertiary UK cancer centres and included all mTNBC patients who received at least one dose of SG as part of the UK compassionate use programme following at least 2 prior lines of chemotherapy (as per the licenced indication). Key endpoints include PFS, OS and safety. Survival was calculated using Kaplan-Meier analysis, comparison of survival curves by log-rank (mantel-cox) test and hazard ratios by Mantel-Haensze test. Statistical analysis was performed on Prism version 9.0 and calculations *P* < 0.05 were considered significant.

## Results

### Patient characteristics

132 patients were included (131 females and 1 male). The median age was 56 years (range 28–91 years).

All patients were ECOG performance status (PS) 0-3 (PS0; 39, PS1; 76, PS2; 16, PS3;1).

75% of patients (99/132) had visceral metastases including 24 patients with central nervous system (CNS) disease and 61 patients with liver metastases (Table 1).

**Table 1:** Patient characteristics of the study population.

Patient Characteristics		
Gender	No. of patients	% of population
Female	131	99.2
Male	1	0.8
Performance status	No. of patients	% of cohort
0	39	29.5
1	76	58
2	16	12
3	1	0.8
Sites of metastasis	No. of patients	% of cohort
Bone	63	48
Visceral	99	75
Liver	61	46
Nodal	102	77
CNS	24	18

SG treatment was administered as 2nd line treatment for 37 patients (28%); 3rd line for 41 patients (31%) and 41% of patients had received 3 or more prior lines of chemotherapy. The median number of prior lines of treatment was 2 (Table 2).

**Table 2:** Number of prior treatment lines in the metastatic setting of patients included.

Number of prior Treatment lines in the metastatic setting	No. of patients	% of population
1	37	28
2	41	31
3	23	14
4	14	11
5	12	9
6	2	1.6
7	2	1.6
8	1	0.8

### Survival analysis

**Fig. 1:**
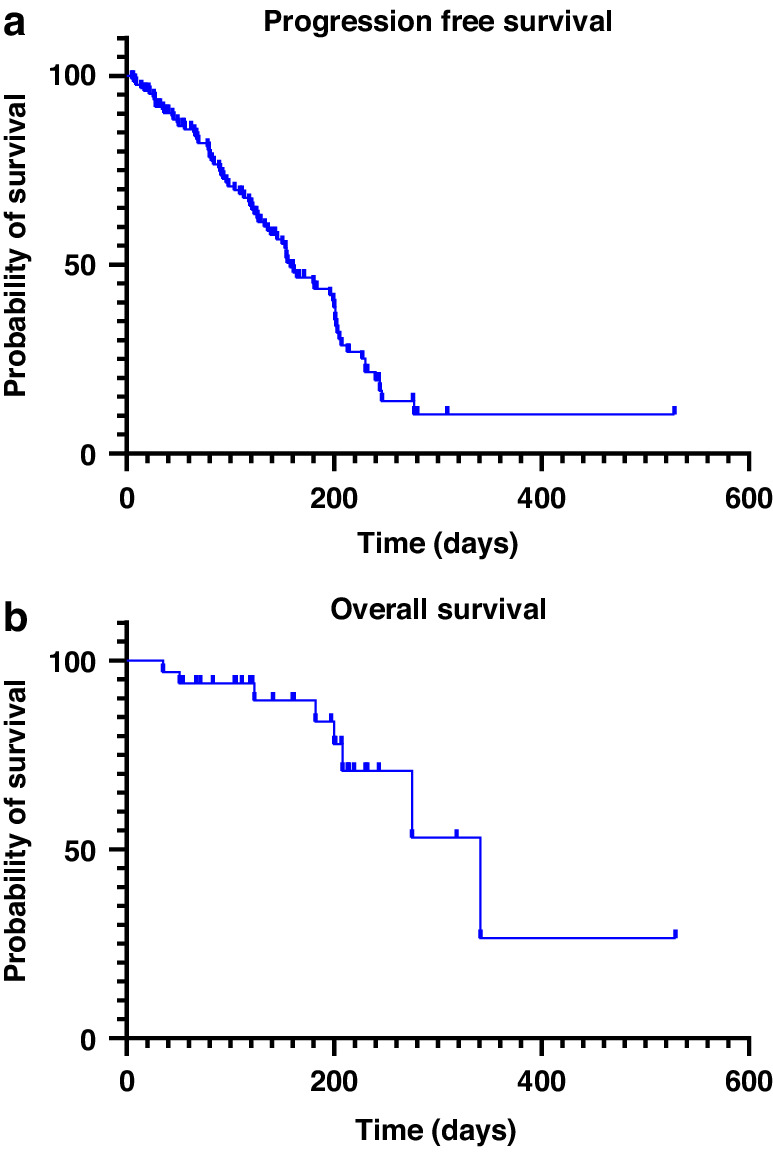
Overall survival of study population. **a** Kaplan–Meier curve of the progression-free survival of study population. **b** Kaplan–Meier curve of the overall survival of study population.

**Fig. 2:**
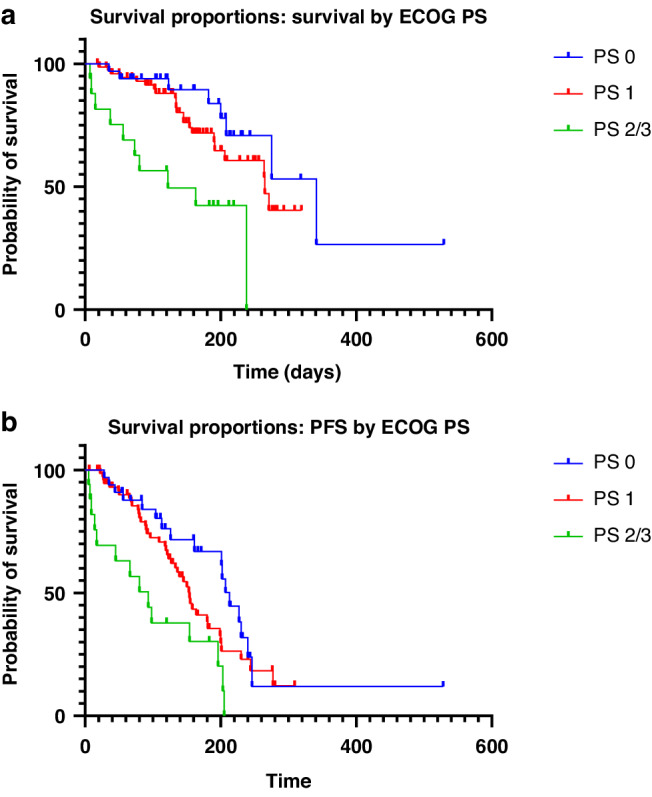
Subgroup analysis of survival by ECOG performance status. **a** Kaplan–Meier curves of the overall survival of ECOG PS 0, ECOG PS 1 and ECOG PS 2 or 3 patients. **b** Kaplan–Meier curves of the progression-free survival of ECOG PS 0, ECOG PS 1 and ECOG PS 2 or 3 patients.

Survival analysis included 126 patients; 6 patients were excluded due to incomplete data. The mPFS was 5.2 months (95% CI 4.5–6.6, Fig. 1a) and the mOS was 8.7 months (95% CI 6.8–NA, Fig. 1b). The mPFS and mOS were significantly different between patients who were PS 0, PS1 and PS2/3 (*p* = 0.0027 and *p* = 0.0015 respectively). The mPFS was 7.0 months (95% CI 5.3–7.9) for PS0 patients, 5.1 months (95% CI 4.2–6.0) for PS1 patients and 3.1 months (95% CI 0.5-6.4) for PS 2/3 patients. The mOS was 11.2 months (95% CI 6.8-NA) for PS0 patients; 8.7 months (95% CI 6.8–NA) for PS 1 patients and 4.0 months (95% CI 1.2–7.8) for PS2/3 patients (Fig. 2a, b).

The mPFS for patients who received 1 or 2 prior treatment lines in the metastatic setting prior to SG was 5.3 months (95% CI 4.4–7.0); the mOS was not reached for this cohort. The mPFS and mOS for patients who received 3 or more prior treatment lines were 5.0 months (95% CI 3.7–6.4) and 8.7 months (95% CI 6.8–11.2) respectively.

**Fig. 3:**
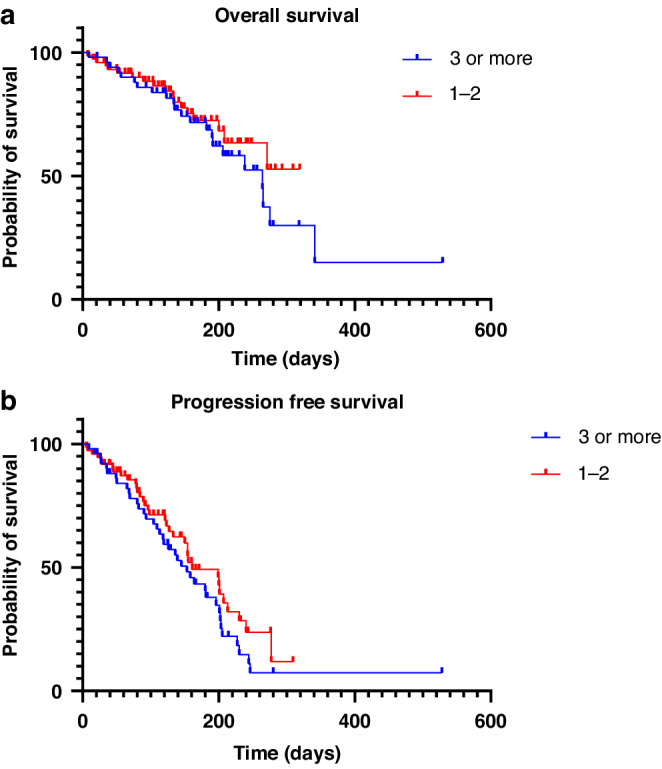
Subgroup analysis of survival by number of treatment lines. **a** Kaplan–Meier curves of the overall survival of patients who received 1–2 and 3 or more prior lines of treatment in the metastatic setting. **b** Kaplan–Meier curves of the progression-free survival of patients who received 1–2 and 3 or more prior lines of treatment in the metastatic setting.

There was no significant difference in mPFS (*p* = 0.21) or mOS (*p* = 0.37) between patients who received 1-2 versus 3 or more prior lines of systemic anti-cancer therapy (SACT) **(**Fig. 3a, b).

### Toxicity analysis

The most common adverse events (AEs) were fatigue (all grade; 82%, G3/4; 14%), neutropenia (all grade; 55%, G3/4; 29%), diarrhoea (all grade; 58%, G3/4, 15%), and nausea (all grade; 38%, G3/4; 3%) (Table 3). SG dose reduction was required in 54% of patients due to adverse events (AEs) and 5% (7/132 patients) stopped SG due to toxicity. The median dose reduction (DR) was 20.3%. 9% of patients (12/132) started treatment at a dose reduction (DR range 10–40%), 31 patients required DR from cycle 2, 9 patients from cycle 3, and 10 patients from cycle 4 or later. The cycle of DR was not specified for 5 patients.

**Table 3:** Summary of adverse events experienced by population (*n* = 132).

Toxicity	Proportion of patients
Nausea	
All Grade	38%
Grade 1	24%
Grade 2	10%
Grade 3	3%
Grade 4	0%
Neutropenia	
All Grade	55%
Grade 1	9%
Grade 2	16%
Grade 3	15%
Grade 4	14%
Diarrhoea	
All Grade	58%
Grade 1	25%
Grade 2	18%
Grade 3	11%
Grade 4	4%
Fatigue	
All Grade	82%
Grade 1	44%
Grade 2	22%
Grade 3	14%
Grade 4	1%

### Subgroup analysis of patients with CNS disease

In our cohort 18% of patients (24/132) had brain metastasis. 8 of these patients were diagnosed with brain metastasis while on treatment with SG. Patients with CNS disease had a mPFS of 5.1 months (95% CI 1.6-6.6); mOS was not reached. The median age for patients with CNS disease was 53 years and the median number of prior treatment lines in the metastatic setting was 2.

Patients with CNS disease who did not receive radiotherapy (RT) at any point (*n* = 12) had a mOS of 2.5 months (95% CI 1.7–NA). 12/24 patients with CNS disease were treated with RT to the CNS before or during treatment with SG. Patients treated with RT had a significantly longer mOS than those not treated with RT (*p* = 0.0025). 13/24 patients with brain metastasis remained on treatment long enough to have brain imaging to assess disease response. 5/13 had a partial response, 1 patient had stable disease and 7 patients had progressive disease. SG ability to cross the blood brain barrier is challenging to dissect from our data as systemic treatment overlapped with radiotherapy in 50% of patients. Furthermore, unlike in the trial setting not all patients had baseline brain imaging to ascertain if CNS disease was present at time of starting SG. A brain staging scan for patients with CNS who did not receive RT was available for 3 patients, all of which reported progressive disease.

## Discussion

This is the first multi-centre national real world study of SG in the UK. Our patient cohort resembled the ASCENT trial in terms of median age, prior lines of treatment and distribution of metastases [16]. We report similar mPFS but a shorter mOS of 8.7 months. The upper confidence interval of mOS could not be calculated as there were not enough later events. One possible contributing factor for the comparatively shorter mOS is our inclusion of patients with a poorer PS, with 13% being PS 2/3 and when selected out, patients who were PS0 had a mOS of 11.2 months which more closely resembles that of the ASCENT population [16].

In ASCENT 71% of the population had received 2 or 3 previous lines of treatment and 29% received 4 or more prior lines. Their median number of previous anti-cancer regimens was 3 including lines in the neo-adjuvant/adjuvant setting. This is similar to our study population: patients had a median of 2 lines of treatment in the metastatic setting. When we compared survival between patients based on the number of prior lines of treatment in the metastatic setting, we found no significant difference. However, mOS was not reached for patients treated with 1 and 2 prior lines of treatment in the metastatic setting with only 10/36 and 8/39 events in each group recorded. This is likely due to a combination of these patients being earlier in their treatment path and living longer plus shorter follow-up time of this cohort.

We included patients with CNS disease in our analysis. The cohort with brain metastasis that was not treated at any point with RT had a very poor mOS of 2.5 months. The exact rationale for withholding RT from these patients is unknown however we can speculate that they were a subgroup of patients with poorer prognoses as they were possibly not considered fit enough to undergo RT.

Data was not collected on granulocyte colony-stimulating factor (G-SCF) use. The Trodelvy summary of product characteristics (SmPC) does not include the use of upfront granulocyte colony-stimulating factor (G-SCF). At present in the UK, upfront G-CSF prescribing is at the discretion of the responsible clinician. Further analysis of use and timing of G-CSF and the impact this has on neutropenia, dose reductions and treatment breaks is warranted.

## Conclusion

This study provides the first real-world experience of SG in mTNBC in the UK, confirming substantial anti-tumour activity in this cohort. The safety profile is consistent with clinical trial experience. Patients with a better performance status had superior survival data. SG efficacy was maintained in patients with CNS disease; however, analysis of larger patient numbers is needed to further assess this subgroup. Our patient cohort was heavily pre-treated as data was collected prior to NICE approval in the UK. Follow-up data in the UK following NICE approval of SG is warranted.

## Data Availability

The datasets generated during this study are available from the corresponding author on reasonable request.
